# The Road Toward Transformative Treatments for Food Allergy

**DOI:** 10.3389/falgy.2022.826623

**Published:** 2022-02-09

**Authors:** Allyssa Phelps, Kelly Bruton, Emily Grydziuszko, Joshua F. E. Koenig, Manel Jordana

**Affiliations:** Department of Medicine, McMaster Immunology Research Centre (MIRC), Schroeder Allergy and Immunology Research Institute, McMaster University, Hamilton, ON, Canada

**Keywords:** allergy (hypersensitive anaphylaxis), immunotherapy, treatment, food allergy (FA), peanut (Arachis hypogaea), immunology

## Abstract

A series of landmark studies have provided conclusive evidence that the early administration of food allergens dramatically prevents the emergence of food allergy. One of the greatest remaining challenges is whether patients with established food allergy can return to health. This challenge is particularly pressing in the case of allergies against peanut, tree nuts, fish, and shellfish which are lifelong in most patients and may elicit severe reactions. The standard of care for food allergy is allergen avoidance and the timely administration of epinephrine upon accidental exposure. Epinephrine, and other therapeutic options like antihistamines provide acute symptom relief but do not target the underlying pathology of the disease. In principle, any transformative treatment for established food allergy would require the restoration of a homeostatic immunological state. This may be attained through either an active, non-harmful immune response (immunological tolerance) or a lack of a harmful immune response (e.g., anergy), such that subsequent exposures to the allergen do not elicit a clinical reaction. Importantly, such a state must persist beyond the course of the treatment and exert its protective effects permanently. In this review, we will discuss the immunological mechanisms that maintain lifelong food allergies and are, consequently, those which must be dismantled or reprogrammed to instate a clinically non-reactive state. Arguably, the restoration of such a state in the context of an established food allergy would require a reprogramming of the immune response against a given food allergen. We will discuss existing and experimental therapeutic strategies to eliminate IgE reactivity and, lastly, will propose outstanding questions to pave the road to the development of novel, transformative therapeutics in food allergy.

## Introduction

Thirty-two million Americans over the age 18 suffer from food allergies, amounting to approximately 11% of the population, and a similar prevalence exists in other developed countries (1–3). The economic impact of food allergies is staggering, estimated at $25 billion in the US annually (4). Each year 200,000 Americans attend emergency departments for food allergy-related anaphylaxis, and a 124% increase in visits was documented between 2005 and 2014 (5, 6). In stark contrast to the impressive social, medical, and economic impact of food allergies, there is a concerning paucity of therapeutic options available for this disease. Indeed, the standard of care for food allergy is allergen avoidance, although oral immunotherapy (OIT) for peanut (PN) has been approved by the FDA in the United States (7). The timely administration of epinephrine upon accidental exposure and other therapeutic options like antihistamines provide acute symptom relief but do not target the underlying pathology of the disease.

The greatest impact in the food allergy field was furnished by a landmark study which provided conclusive evidence that the administration of PN to infants 4–11 months old dramatically reduces the incidence of PN allergy (8). Yet, this major advancement leaves the millions of patients diagnosed with food allergies worldwide unprotected. Clearly, one of the greatest remaining challenges is whether established food allergy can be fundamentally mended. This challenge becomes even more pressing for allergies such as those to PN, tree nuts, fish, and shellfish which are lifelong in most patients (7, 9, 10). In this context particularly, a “disease-transformative” treatment would require the restoration of an active non-harmful immune response to foods, known as tolerance. We define a “disease-transformative” treatment as one that successfully alters the underlying disease mechanisms permanently.

In this review we will focus on the immunological mechanisms which maintain lifelong food allergies and are, consequently, those which must be dismantled or reprogrammed to restore health. We will also discuss allergen immunotherapy (AIT) and approaches currently under investigation purported to deviate the immune response against food allergens away from a pathogenic response. A distinctive feature of food allergies is that the clinical phenotype is largely, if not exclusively, mediated by IgE. Therefore, the discovery of “disease-transformative” treatments requires a comprehensive understanding of the cellular and molecular mechanisms that underlie the lifelong capacity to produce allergen-specific IgE. Such understanding shall pave the road to novel therapeutics in food allergy.

## The Immunobiology Underlying The Persistence of Food Allergy

Allergic sensitization begins with the disruption of homeostasis at mucosal sites or the skin resulting in the release of alarmins such as IL-33, IL-25 and TSLP (11). The mechanisms of homeostatic disruption which underly allergic sensitization have been thoroughly reviewed elsewhere (11). This breach of homeostasis leads to the differentiation of allergen-specific CD4^+^ Th2 cells, T follicular helper (Tfh) cells, short-lived plasma cells, and memory B cells (MBCs), all of which contribute to IgE generation and clinically active food allergy (12, 13). Serum IgE levels are extremely low compared to other isotypes and IgE has a half-life of approximately 3 days in humans, a rapid turnover rate in comparison to other immunoglobulin isotypes (14–16). Evidence for a role of long-lived plasma cells replenishing these IgE titres, and in the persistence of food allergy has long been disputed (12, 17). In a mouse model of PN allergy, allergen avoidance results in a decline in allergen specific IgE titres, which are undetectable by 6 months post-sensitization, indicative of a lack of long-lived antibody secreting cells (18). Instead, IgE titres are transiently maintained following sensitization by short-lived IgE^+^ plasma cells. In the peripheral blood of PN-allergic patients, IgE^+^ plasma cells have an immature transcriptional program characterized by the upregulation of MHC and low-affinity IgE receptor (CD23) and downregulation of plasma cell survival genes (19, 20). Further, in PN-allergic mice avoiding PN, IgE^+^ plasma cells have a half-life of approximately 60 days (18). Once bound to mast cells, IgE-mediated mast cell degranulation upon challenge has a half-life of approximately 70 days in mice prior to any subsequent allergen exposure, indicating that undetectable serum IgE does not preclude clinical reactivity upon allergen exposure (18). Similar evidence exists in humans allergic to galactose-α-1,3-galactose, whose serum allergen-specific IgE titres declined when avoiding the allergen (tick bites) (21). This short-lived nature of IgE^+^ plasma cells is also evident in humans suffering from seasonal allergic rhinitis, wherein the IgE titers decline off-season and rise during on-season (22). Arguably then, long-lived plasma cells do not retain long-lived IgE memory. Despite the accumulation of evidence that IgE responses in both mice and humans are transient, there are some observations which do not yet have a conclusive explanation. In a model of chronic house dust mite exposure, a population of long-lived IgE-expressing cells were detected in the bone marrow, suggesting that in some contexts long-lived IgE^+^ PCs may be generated (23). Thus, to definitively rule out the role of long-lived IgE^+^ PCs in PN allergic individuals further research is required. This is a technically challenging endeavor as the rate of accidental exposure to PN is 12.4% annually and these cells would likely be extremely rare if they do exist (24). Nonetheless, the possibility exists that some long-lasting IgE^+^ PCs may reside in mucosal sites or in the bone marrow of these allergic individuals, but limitations in acquiring these samples for study have precluded their detection.

IgE titres are rapidly replenished upon activation of MBCs following a secondary allergen exposure. However, IgE^+^ MBCs are extremely rare or non-existent in humans and are, therefore, not considered relevant to the persistence of food allergy (20, 25, 26). The recent identification of IL-13-expressing Tfh cells (Tfh13) demonstrated that Tfh13 cells promote IgE^+^ B cell survival in germinal centers (GC), leading IgE^+^ B cells to preferentially differentiate into plasma cells (27–29). In contrast to IgE^+^ MBCs, non-IgE MBCs have been shown to maintain long-lived food allergy, particularly IgG1^+^ MBCs (17, 25, 30). Although IgE^+^ B cells participate transiently in GCs, IgG1^+^ B cells persist within GCs and differentiate into affinity matured MBCs (17, 30). Upon secondary allergen exposure, IgG1^+^ MBCs rapidly undergo class-switch recombination (CSR) and differentiate into IgE^+^ plasma cells to maintain IgE responses (30, 31). In humans, all upstream isotypes are also clonally related to IgE, indicating that other isotypes may also be capable of holding allergic memory (26, 32).

Assessing the requirements for secondary responses to allergens is critical to understand the persistence of food allergy and, consequently, to develop novel therapies. MBC reactivation leading to IgE production is strictly dependent on CD4^+^ T cells and IL-4, specifically through IL-4Rα signaling, which is a fundamental requirement for IgE CSR (33). Studies in mice indicate that IgE CSR requires Tfh cell-derived IL-4 during primary responses, though the source of IL-4 during secondary responses, particularly in humans, remains unclear (34, 35). Whether the requirement of CD4^+^ T cells during secondary responses is fulfilled by Tfh cells, Tfh13 cells or Th2 cells remains to be elucidated. IL-13 also signals through IL-4Rα and the role of Tfh13 cells in IgE responses indicates that IL-13 may play a non-redundant role in high-affinity, anaphylactic IgE production (27, 29). Allergen-specific Th2 cells have also been implicated in the pathogenesis of food allergy. Th2 cells are generally defined by their secretion of Th2-polarized cytokines such as IL-4, IL-5, and IL-13 and high expression of GATA3, though many diverse subpopulations have been characterized with distinct phenotypes (36). Their role in IgE production remains unclear, though they do contribute to late phase inflammation (12). Although CD4^+^ T cells are fundamentally required to initiate secondary B cell responses, the role of memory CD4^+^ T cells in this process remains unclear.

The importance of memory CD4^+^ T cells in the recall response may be questioned by data generated from adoptive transfer studies suggesting that naïve CD4^+^ T cells and allergic B cells are sufficient to re-establish peanut-specific IgE production and clinical reactivity (37). It is possible that allergen-specific MBCs may be capable of polarizing naïve CD4^+^ T cells toward a Th2 phenotype during recall responses. Nonetheless, it is important not to disregard the possible contribution of memory CD4^+^ T cells to food allergy persistence, particularly in an environment where they compete with naïve CD4^+^ T cells. Given that Tfh cells are critical drivers of IgE CSR, memory Tfh cells may also play a role in secondary IgE responses, though this remains to be determined. Furthermore, a subpopulation of terminally differentiated, allergen-specific “Th2A” cells has been identified exclusively in allergic individuals which expand upon allergen exposure, suggesting their implication in allergic pathology (38). Thus, MBCs are likely not the only cells that contribute to recall responses, and hence, the persistence of food allergy.

## Terminology For Food Allergy Treatments

Generally, an individual is referred to as sensitized when allergen specific IgE is detected in their serum, or they have a wheal/flare reaction to a skin test (39) (Table 1). Sensitization does not necessarily imply that the patient will experience an allergic reaction upon allergen exposure. For example, patients with severe atopic dermatitis often have high food-specific IgE titres, but do not experience symptoms upon ingestion (40). Also, food specific IgE may be detected in some non-allergic individuals (8, 41). It is not yet clear, in either context, why these IgE antibodies do not cause allergic symptoms, though one proposed explanation is that food specific IgE in symptomatic patients may have higher affinity for the food allergen than in non-allergic individuals. Among those who are sensitized, individuals who experience an allergic reaction upon ingestion of an allergen are referred to as being clinically reactive, though this is often used interchangeably with being “allergic.” A diagnosis of food allergy typically requires an assessment of multiple factors, including a history of clinical reactivity, the signs and symptoms experienced, along with measures of sensitization.

**Table 1:** Clinical terminology defined.

**Term**	**Definition**
Sensitization	The presence of allergen specific IgE, or reactivity to allergen in a skin prick test.
Clinical reactivity	Symptoms of an allergic reaction following allergen ingestion.
Desensitization	Increase in allergen consumption threshold in allergic patients while on therapy.
Remission (Sustained unresponsiveness)	Absence of clinical reactivity to an ingested food allergen at some timepoint after therapy has been discontinued and allergen has been avoided. Most subjects do not experience sustained unresponsiveness following OIT, but those who do are typically instructed to introduce allergen *ad libitum* into their diet.
Immunological tolerance	The immune response to ingested food antigens in healthy individuals that allows for ingestion of foods without adverse reactions.

As trials for various therapies have progressed, primarily allergen immunotherapies, a series of terms have arisen to define the possible outcomes for allergic patients. These terms generally refer to clinical phenomena, where the mechanistic immunological foundation of the terms remain elusive. The terms outlined here are an extension of definitions provided by Burks et al. (42). The success of a therapy is typically assessed using an oral food challenge, feeding allergen in increasing amounts in a controlled clinical setting. A patient is referred to as **desensitized** if their threshold for allergen consumption without clinical reactivity has increased to a dose defined uniquely by each study. Desensitization is associated with decreased skin test size and decreased levels of sIgE, but it is common for a desensitized patient to retain a certain level of sIgE over the course of therapy (43). It has become clear that desensitization does not imply modification of the underlying mechanism of disease for most patients. Indeed, upon cessation of therapy, i.e., allergen consumption, most desensitized patients relapse into clinical reactivity (44–46). The small proportion of patients who remain unresponsive to oral food challenge following cessation of treatment is referred to as having achieved **sustained unresponsiveness**, or **remission**, and patients are generally recommended to incorporate the allergen into their diet. However, there is no consensus for how long an individual must remain unresponsive to warrant the title. Studies have used timeframes from 2 weeks up to 1 year (44–46). After cessation of therapy the number of patients who relapse increases over time. Further, the protection from clinical reactivity in patients in remission is thought to be maintained by allergen consumption. This has led to the hypothesis that remission may represent a transient state of protection while the underlying mechanisms of disease persist.

Remission is often used as an alternative to the word “tolerance,” which is a contentious concept. In the clinical context, drug tolerance refers to a decrease in the efficacy of a drug over the course of repeated administrations. This observation is similar to desensitization- the threshold of allergen required to induce a clinical reaction is elevated. In the immunological context, tolerance refers to the process by which the immune system is educated into non-responsiveness to self-proteins (e.g., central tolerance) or to innocuous environmental antigens (e.g., foods) (47–49). The induction of immune tolerance is achieved when antigens are ingested in the absence of concomitant homeostatic perturbations. As far back as 1911, Wells and Osborne made the seminal observation that guinea pigs fed corn were protected from sensitization and anaphylaxis against zein, a corn protein (50). **Immunological tolerance** is the typical immune response to orally ingested antigens that enables lifelong routine consumption of foods, regardless of the length of time since food consumption. Rather than being mediated by an elimination or anergization of food-specific cells, immunological tolerance to foods appears to be an active immune response characterized by the presence of T regulatory cells and circulating food-specific antibodies of varying isotypes, except IgE (51). Clearly, for the majority of allergic patients, remission does not amount to immunological tolerance. In fact, it is not clear whether true immunological tolerance can be induced in allergic patients and, further, it is important to question whether this should be the objective of a treatment. Future research is needed to understand whether there exist healthy states somewhere between remission and immunological tolerance which confer a lifelong absence of clinical reactivity and which may be a more attainable goal than true immunological tolerance (52). One such example may be the immunological state achieved in individuals who outgrow food allergies. Outgrowth occurs in only about 20% of PN allergic patients but is much more common in milk and egg allergy which is typically outgrown early in life (49). A detailed phenotypic characterization of outgrown allergy compared to naturally tolerance and persistent allergy has not yet been performed. This research is crucial to understand whether outgrowth is a state of true tolerance or a non-reactive state between remission and tolerance. The former would suggest that true immunological tolerance is a reasonable goal of therapy, while the latter could characterize a state which may be a more suitable target for therapy.

Assuming this knowledge gap, the ideal treatment for food allergy would be one where the therapy is temporary, yet its effects persist permanently regardless of whether allergen is consumed. Such a therapy either eliminates the ability to produce allergen-specific IgE, or permanently renders allergen-specific IgE inert. Since the ability to regenerate allergen-specific IgE is maintained by immune memory, any such therapy must either deplete or reprogram the memory compartment such that allergen-specific IgE is not regenerated.

## Current and Investigative Treatments For Food Allergy

The only FDA-approved treatment for food allergy, particularly PN allergy, is Palforzia, an orally administered PN OIT tablet (53). OIT is a form of AIT that involves the daily oral consumption of allergen. A standard dose escalation protocol for OIT begins with an in-clinic supervised graded challenge to determine a well-tolerated starting dose. This is followed by gradual up dosing generally over 6–12 months until reaching a maintenance dose (300–4,000 mg) that is continued for a year or longer. Most patients on OIT become successfully desensitized (53–55). Sixty-seven percent of patients on Palforzia, aged 4–17, achieved desensitization at the end of the study period, compared to 4% on placebo treatment (53). Despite the significant protection offered by OIT, a systematic review found that OIT regimens for PN resulted in an increase in serious adverse events (AEs), defined as life threatening or requiring urgent medical intervention and/or hospitalization, as well as an increase in epinephrine use, compared to allergen avoidance (55). An additional layer of complexity is that approximately 30% of patients under the age of 18 are allergic to multiple foods (56). However, a study by Bégin et al showed that OIT for up to five different allergens had a safety profile that is similar to single allergen OIT with mild reactions reported in 3.4 vs. 3.7% during updosing of multi-food and single allergen OIT respectively, and only two severe reactions requiring epinephrine in both scenarios (2/25 on multi-food OIT and 2/15 on single allergen OIT) (56). While multi-food OIT required a longer dose escalation protocol, participants in this study ultimately reached an equivalent fold increase in dose per food.

Clinical trials have demonstrated that the risk of AEs with AIT is in part dependent on the route of allergen administration. Alternative routes include epicutaneous immunotherapy (EPIT), sublingual immunotherapy (SLIT), and subcutaneous immunotherapy (SCIT). These options result in reduced rates of adverse reactions and, for SLIT, increased patient compliance (57–62). However, the efficacy of desensitization is lower, particularly for EPIT. Peptide immunotherapy is another approach to minimize AEs by replacing whole allergens with short allergen peptides, which have a reduced capacity to crosslink IgE on the surface of mast cells and basophils (63, 64). Preliminary findings from a clinical trial for PN allergy demonstrated no serious AEs (63). Regardless of the route of administration, the assessment of efficacy and safety of AIT is complicated by variations in methodology. For instance, the dose of allergen administered during the escalation and maintenance phases is variable across clinical trials, as well as the duration of treatment and the form of allergen (e.g., powder, pill, cookie) (65–67). One substantive explanation for the high rate of serious AEs may be the high dose of allergen. In this regard, a clinical trial where a low maintenance dose of allergen (equal or <300 mg) and long updosing was used found that 74.2% of patients were protected at the time of the oral challenge after 16 months vs. 16% placebo and no epinephrine was required for AEs (68). To conclude, reducing the rate of serious AEs without compromising efficacy is critical for the widespread use of AIT as a treatment of food allergy.

The mechanism by which AIT induces desensitization remains to be fully elucidated. Clinical studies have demonstrated changes in the composition of the humoral response, in particular an increase in PN-IgA and PN-IgG4 as well as a decrease in PN-IgE (44, 69–71). Increased PN-IgA is thought to play a role in the induction of tolerance, potentially through targeted uptake of allergen (72, 73). IgG4 is thought to contribute to desensitization through several mechanisms, one of which is its role in mast cell and basophil reactivity, as basophils and mast cells passively sensitized with plasma containing PN-IgG4 could not be activated after PN stimulation (74). PN-IgG4 has been postulated to facilitate desensitization through its binding to the inhibitory IgG receptor (FcγRIIb), thus suppressing IgE signaling (75–77). Furthermore, circulating PN-IgG4 antibodies can bind and sequester allergen decreasing the recognition by IgE antibodies. The induction of PN-IgG4 during AIT appears to be a result of IL-10-producing regulatory T or B cells, which are increased in desensitized individuals (78–80). Specifically, *in vitro* studies have shown that IL-10-producing FoxP3^+^ Treg cells preferentially induce IgG4 secretion in B cells (80). The expansion of Tregs may be mediated by desensitized intestinal mast cells, which were recently found to secrete IL-2 and induce FoxP3 expression in CD4^+^ T cells in a murine model of AIT (81). Moreover, Tregs isolated from desensitized OIT patients significantly supressed T effector cell proliferation to PN compared to Tregs from non-desensitized patients (82). However, whether increases in Tregs have a causative role on OIT beneficial clinical outcomes remains disputed (83). Despite these immune modulating effects, analysis of PN-specific CD4^+^ T cells on OIT has uncovered a subset of pathogenic Th2 cells termed ‘Th2A' cells that also decrease but remain persistent after therapy (38). Furthermore, recent evidence has shown that Th2A-like cells on PN-OIT had a suppression of Th2 genes, as exhibited in anergic states. However, Tfh cells, which may have a major role in CSR during secondary responses, are unaffected by PN-OIT (83). Likewise, PN-specific B cells transiently increase at the beginning of OIT before declining as therapy continues, but are not eliminated (26, 84). It would stand to reason to argue that the persistence of pathogenic cells following AIT undermines the establishment of a long-lasting non-reactive state.

Various experimental approaches are focused on the use of biologics, either alone or as an adjunct to AIT (Table 2). For example, anti-IgE monoclonal antibodies (such as omalizumab), which sequester free IgE, have been investigated in food allergy as either a monotherapy or in combination with OIT (85–87). Omalizumab administered during the dose-escalation phase of OIT decreased the time required to reach the maintenance dose (88, 89). For instance, 80% of participants pre-treated with omalizumab were able to consume 250 mg of allergen on the first day of desensitization, whereas only one of eight participants without omalizumab tolerated this amount (89). Other adjunct therapies to AIT aim to alter the balance of the Th response either through shifting away from Th2 polarization, eliminating the Th2 compartment altogether, or inducing a tolerogenic response through the induction of Tregs. For example, TLR4 and TLR9 both lead to the secretion of type 1 cytokines and the development of a Th1 immune response (90). Disarming T cells involved in allergy is being investigated with AIT plus Abatacept, an IgG Fc domain fused to cytotoxic T lymphocyte antigen-4 (CTLA-4), a co-stimulatory molecule required for T cell activation (91). Thereby, Abatacept aims to block T cell co-stimulation during AIT, leading to T cell anergy and/or Treg induction. Most of these approaches have demonstrated efficacy in preclinical murine studies and those denoted by an ^*^ in Table 2 are being tested in ongoing clinical trials for PN allergy (NCT02402231, NCT01781637, NCT04872218, NCT03682770, NCT03463135).

**Table 2:** Current treatments under investigation for food allergy.

**Strategy**	**Approach**	**Description**	**Model**	**Immunological changes**
Elimination of Th2 components	Dupilumab (19)*	Anti-IL-4Rα Monoclonal antibody- dually inhibits IL-4 and IL-13	Ovalbumin (OVA) Allergy ↓ Anti-IL-4Rα (i.p.)	Decreased PN-IgE levels Inhibited anaphylaxis
	Omalizumab (92)*	Anti-IgE monoclonal antibody	PN Allergy ↓ Anti-IgE (i.p.) plus OVA (oral)	Reduced anaphylactic response
	Etokimab (93)*	Anti-IL-33 antibody - inhibits the alarmin IL-33	Preclinical Trial PN Allergy ↓ Anti-IL-33 (i.v.)	Decreased IL-4, IL-5, IL-9, IL-13 and ST2 CD4^+^ T cells Decreased PN-IgE Increased successful oral food challenges
Shifting Th2 to Th1	Glucopyranosyl Lipid A (94)*	Toll-like receptor 4 Th1 Adjuvant	PN Allergy ↓ OVA plus GLA (s.l.)	Inhibited anaphylaxis
	CpG (95)	Toll-like receptor 9 Th1 Adjuvant	PN Allergy ↓ PN plus CpG (i.n.)	Increased IL-10 and IFN-γ and decreased IL-13 Reduced anaphylactic response Increased PN-specific IgG2c and mucosal IgA
Induction of T regulatory cells	Transforming growth factor (TGF)-β (96)	Immunoregulatory cytokine	OVA plus TGF-β (oral) ↓ OVA Allergy	Decreased OVA-IgE and OVA-IgG1levels
	Abatacept (91)	Fc region of IgG1 fused to anti-Cytotoxic T lymphocyte antigen-4	OVA Allergy ↓ OVA plus Abatacept (s.c.)	Suppressed airway hypersensitivity Decreased OVA-IgE levels Decreased Th2 cells
	IL-2 (97)	Anti-IL-2 monoclonal antibody IL-2/anti-IL-2Ab complex	Milk Allergy ↓ OVA (s.l.) plus IL-2 (i.p.)	Decreased IL-5 Increased IL-10 and TGF-β Decreased IgE levels

The * symbol denotes treatments currently in clinical trials.

The table above describes biologics that are currently under investigation for the treatment of food allergy. The table includes overarching strategies of treatments, examples of biologics and their description. The fourth column describes models in which the biologics have been tested thus far. It includes (1): the allergen used; (2): whether the treatment or allergic sensitization occurs first (depicted by the arrow); and (3): the route in which treatment was applied. Intraperitoneal injection is denoted as i.p., intravenous injection is denoted as i.v., intranasal is denoted as i.n., subcutaneous injection is denoted as s.c. and sublingual is denoted as s.l. Lastly, the fifth column describes the associated immunological changes reported using the models in the fourth column.

A variety of vaccine approaches currently explore their potential to skew the Th2 allergic program toward Th1 in PA. For instance, lyosomal-associated membrane protein-1 (LAMP-1) DNA vaccines are vectors encoding both LAMP1 and immunodominant allergens. Transcribed and translated allergens are directed to the lysosome and internally linked to the lysosomal membrane. Thereby, unlike AIT, no allergen is released into circulation, avoiding unwanted immune recognition by mast cells and basophils (98). The LAMP-Vax platform is associated with the induction of IFN-γ and allergen-specific IgG2a (99). A clinical trial of a multivalent (Ara h1, h2, h3) lysosomal associated membrane protein DNA plasmid vaccine is currently underway (ASP0892). In addition, a virus-like particle (VLP) vaccine, which displays single PN allergens, has shown promise *in vivo* (100). Indeed, mice immunized with the VLP vaccine after sensitization had increased levels of PN-specific IgG2a and IgG2b, and were protected from anaphylaxis upon allergen challenge, in a manner dependent on the inhibitory FcγRIIb. Lastly, a new vaccine composed of nanoscale oil-in-water emulsion (nanoemulsion) adjuvanted allergen has been tested intranasally (101). In murine studies this vaccine suppresses the secretion of Th2-polarizing alarmins and cytokines, while increasing IL-10 and Tregs through an IFN-γ dependent mechanism and subsequently decreasing reactivity upon allergen challenge (101). Clinical trials of these vaccination strategies are ongoing or being initiated.

The clinical outcome of strategies that aim to repolarize or, to an extent, suppress the immune response against food allergens is not known. However, studies examining the immunological impact of AIT have revealed that, at least for T cells, subsets that seem to have a critical role in IgE regeneration are relatively unaffected by the treatment. This would argue that any strategy that is not able to delete these subsets or to dismantle allergen-specific memory will have a limited, although not irrelevant, clinical impact.

## Future Avenues For Food Allergy Treatment

Adaptive immunity evolved to be long-lasting and specific, enabling a rapid recall response to prevent any single pathogen from eliciting malady twice. For example, primary infection with varicella-zoster virus induces humoral and cellular memory which protects against viral disease despite inevitable re-exposures and the virus itself establishing latency in ganglionic neurons; only when immunological memory becomes compromised (e.g., aging, drug-induced immunosuppression, etc.) will varicella-zoster virus establish secondary illness (shingles). However, in at least two instances – allergy and autoimmunity – immunological memory perpetuates disease, rather than protecting against it. This inherent durability of immunological memory is the greatest challenge faced in efforts to therapeutically reverse, reprogram, or cure food allergy.

Perhaps the most dramatic impact on allergy in humans is through a total factory reset of the immune system. Following hematopoietic stem cell transplants (HSCTs) for the treatment of malignant or non-malignant diseases unrelated to allergy, over 90% of allergic recipients lost allergen-specific IgE reactivity when receiving a transplant from a non-allergic donor (102). Two years post-transplant, no recipients had regained allergic reactivity. HSCTs are preceded by total body irradiation or chemotherapy-based conditioning, which depletes cells of hematopoietic origin including memory T and B cells; however, long-lived plasma cells exhibit radiation resistance (103). The rapid loss of allergen-specific antibody titers following myeloablation and HSCT reaffirms observations in animal modeling suggesting that long-lived plasma cells do *not* maintain lifelong food allergies (18, 30). Thus, the pathway which replenishes short-lived plasma cells is the critical therapeutic target. Certainly, HSCTs are only warranted in the most extreme scenarios, but the success in eliminating allergic reactivity without relapse begs the question: what strategies can be employed for the *targeted* removal or disruption of pathogenic lymphocytes underlying the maintenance of food allergy (Table 3)?

**Table 3:**
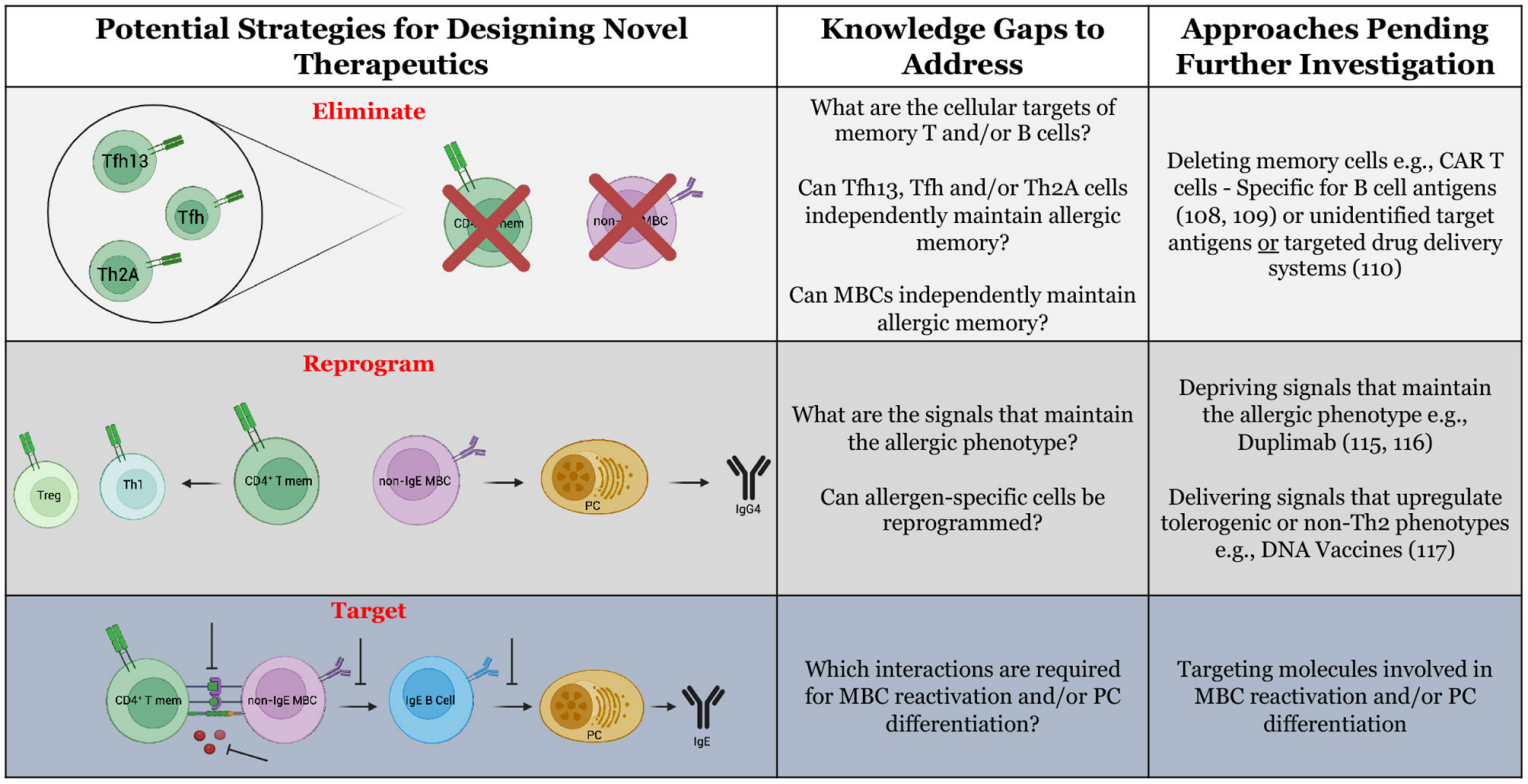
Potential strategies for the design of novel therapeutics for food allergy.

*This table describes potential strategies to develop novel therapeutic for the treatment of food allergy, the knowledge gaps that must be addressed as well as some approaches that require further investigation*.

Innovative food allergy treatments may be inspired by the successes of targeted cancer immunotherapies. Treatment of food allergy and cancer possess a common goal of eliminating cells of a certain specificity/phenotype. The advent of antigen receptor engineering has enabled the delivery of T cells or NK cells that express antibody-like receptors called chimeric antigen receptors (CARs) which are specific to neoantigens or tumor-associated antigens (104, 105). The inclusion of costimulatory domains in the CARs overrides the need for secondary signals provided by antigen-presenting cells (106). Upon recognition of cell surface antigen through their engineered receptors, the cells execute their intrinsic cytotoxic functions resulting in destruction of malignant cells. Theoretically, CAR-T cells could be engineered to interact with allergy-associated cell surface molecules on T and B cells. A potential T cell target is the prostaglandin D_2_ receptor, CRTH2, which is expressed by allergen-reactive Th2 cells (38). An equivalent marker unique to allergen-specific B cells has not yet been defined. Although CRTH2 and other molecules can be upregulated by allergic cells, the inherent redundancy of the immune system makes it unlikely that these markers are unique to allergen-specific cells. Therefore, one consequence of using CARs built for allergy- “associated” molecules may be adverse off-target effects, such as excessive cytokine production and tissue damage (107).

An optimal approach for targeted allergy immunotherapy would exploit antigen receptors (TCRs and BCRs) specific to the allergen(s) of interest. Proof-of-concept studies have demonstrated success in engineering CAR-T cells to express B cell antigens (in place of the antibody-like binding domain) upstream to costimulatory domains. This construct was termed B cell-targeting antibody receptors (BARs) (108). Delivery of OVA-specific BAR-Treg cells to OVA-allergic mice reduced the severity of anaphylaxis upon systemic challenge (109). While promising, the mechanism by which this protection is achieved and its longevity remain unknown. Targeting allergen-specific T cells in this manner would be far more challenging, as it would require expression of multiple different MHC:peptide complexes to encompass each of the immunodominant peptides for a given allergen. As opposed to cellular engineering, drug conjugation may be an alternative approach to target allergen-specific cells. Some cancer therapies utilize a “warhead” strategy wherein chemotherapeutic drugs are conjugated to monoclonal antibodies, promoting targeted drug delivery (110). This strategy could be adopted to allergy whereby whole allergens or allergen peptides are conjugated to cytotoxic drugs. One potential limitation of approaches involving engineered allergen expression or delivery of allergen-drug conjugates is that there are often high levels of circulating allergen-specific antibodies in allergic patients, which may severely limit bioavailability. As well, delivery of whole allergen proteins may cross-link IgE on mast cells or basophils, resulting in unintended allergic reactions, though this may be ameliorated by co-administration of omalizumab.

Furthermore, it may be possible to alter or reprogram the phenotype of allergen-specific cells in lieu of their physical elimination. The potential ability to induce cellular reprogramming originates from the concept of plasticity. Beyond the description of a terminally-differentiated Th2A cell phenotype, the plasticity of allergen-specific cells is not well understood (38). The ability for allergy outgrowth implies some degree of functional plasticity, though it is unclear why outgrowth occurs so infrequently in peanut, tree nut, fish, and shellfish allergies in comparison to milk and egg allergies (9, 111, 112). If allergen-specific cells are functionally plastic, therapeutics could be designed to deprive cells of signals that maintain pathogenicity. For example, IL-4 is critical for the induction of allergy, including Th2 polarization and IgE production (113, 114). In recent work with PBMCs from peanut-allergic patients, we have shown that IL-4 deprivation through therapeutic IL-4Rα blockade dampens the IL-4-responsive phenotype in allergen-reactive MBCs and upregulates IFN-γ production (19). Similarly, in a murine asthma model, use of a small molecule inhibitor of STAT6 (involved in IL-4/IL-13 signaling) reversed airway hyperreactivity (115). Alternatively, it may be possible to design therapeutics which deliver signals to actively upregulate tolerogenic or non-Th2 phenotypes. The use of a DNA vaccine is one such example, as previously mentioned. Delivery of allergen-encoding plasmid DNA provides strong Th1 signals (IFN-γ and IgG2a) which may aid to counteract the Th2 dominant signature (116). An ideal “disease-transformative” DNA vaccine would consist of a single dose that could reprogram the immune response, though it is not clear at this early stage whether this is possible with this strategy.

Lastly, it is evident that we have reached a ceiling as to what AIT can achieve as a monotherapy (117); however, the exhaustive number of AIT studies have provided a well-defined regimen for supervised allergen exposure, which may be applicable in combination therapies. Biologics for the treatment of allergic diseases have shown efficacy when administered as a monotherapy. For example, dupilumab monotherapy in atopic dermatitis, asthma, and chronic rhinosinusitis with nasal polyposis is highly effective in ameliorating disease score and IgE titers (118–120). These conditions, however, are distinct from allergy in that IgE specificity is broad and production is perpetual. This is in contrast to food allergy wherein IgE titers are highly specific to the eliciting allergen(s) and is only produced upon allergen exposure. Thus, dupilumab monotherapy in food allergy will likely prevent IgE production upon accidental exposures but will forgo the benefit of allergen-specific cell reprogramming. In those that attained clinical remission from atopic dermatitis via dupilumab, skin-resident Th2A cells persisted (121). Deliberate activation of allergen-specific cells in a tolerogenic or non-Th2 context (via biologics or other therapeutics) is more likely to facilitate reprogramming.

Currently, the development of novel, efficacious therapies is limited by an incomplete understanding of the underlying immunological mechanisms. To rationally design novel therapeutics, we propose that the following three questions must be addressed from a basic immunological standpoint:

*1) What are the fundamental requirements for the perpetuation of allergic disease?* It remains unclear whether allergen-specific memory T or B cells can independently maintain allergic disease. Likewise, the relative contributions of Th2A, conventional TFh, and TFh13 cells in the regeneration of IgE responses remains unknown. Investigation of these issues will be critical to determine whether future therapies should target one or more cell types. For example, if Th2-polarized T cells are the minimal requirement, then therapeutic targeting of MBCs would be insufficient. These investigations should extend even beyond adaptive immunity, to address whether conditioning of innate cells (trained immunity) is capable of re-establishing IgE responses (122, 123).

*2) What molecular cues are necessary to replenish the short-lived IgE PC pool?* B cell activation occurs through intricate interactions with secreted and membrane-bound molecules. The interactions involved in MBC reactivation and PC differentiation, however, remain elusive. A more complete understanding of these processes is critical for the development and use of biologics. As of 2012, clinical trials employing biologics for eight different targets (IgE, IL-5, IL-4, IL-13, IL-17, IL-9, GM-CSF, TNFα) were already underway for the treatment of asthma (124). Nearly 10 years later, biologics are being trialed for only four targets (IgE, IL-4R, IL-33 and Glucopyranosyl Lipid A) in food allergy.

*3) Is the allergic phenotype plastic?* Despite several indications that the allergic response may be reprogrammed, it has not been well established whether the induction of non-Th2 phenotypes arise from *de novo* responses or a reprogramming of the existing allergen-specific memory cells. This distinction will help to inform whether a non-Th2 population can effectively outcompete pathogenic allergen-specific cells or if persisting pathogenic cells will eventually undermine therapeutic reprogramming. If it is possible to reprogram cells out of a pathogenic phenotype, it will be critical to determine if there is a risk of relapse. Without the ability to reprogram cells, lifelong treatments may be required, as appears to be the case for AIT monotherapy.

## Concluding Remarks

In contrast to cancer and chronic inflammatory diseases such as rheumatoid arthritis and inflammatory bowel disease and, even, asthma, the advent of novel treatments for food allergy has lacked appallingly behind. Indeed, the latest FDA-approved treatment for food allergy is an old friend, namely oral immunotherapy, which is clearly not a “transformative” therapy for most patients. The reason for this state-of-affairs is seemingly clear: the development of novel therapeutics is directly related to the state of fundamental knowledge of a given disease process. In this regard, the last decade has unearthed consequential advances in our understanding of the basic cellular and molecular mechanisms underlying IgE biology and the persistence of food allergy. Particularly, whether the long-lived capacity to generate IgE entirely lies within the IgG1 memory compartment. Expectedly, a number of biologics and novel approaches are currently being trialed, and there is little doubt that more will emerge during the present decade. Still, as intimated in the previous section, much remains to be learned to develop treatments that are precise and specific, i.e., target-precise and allergen-specific. As we have noted elsewhere, the ever-increasing application of technologies that deliver formidable datasets in both human tissues and mouse systems will help to delineate complex inter- and intracellular pathways, thus revealing the astounding phenotypic diversity and plasticity of the food allergic response. This will lead to a greater appreciation of the genetic signatures and subpopulations that define disease states and, as a result, usher the discovery of personalized therapeutics.

## Author Contributions

AP, KB, EG, JK, and MJ all wrote and edited the manuscript. All authors contributed to the article and approved the submitted version.

## Funding

The research in MJ's lab was supported by funds from Food Allergy Canada, the Walter and Maria Schroeder Foundation, the Michael Zych Family, and the Canadian Asthma, Allergy, and Immunology Foundation (CAAIF). KB holds a Canada Graduate Scholarship. AP holds an Ontario Graduate Scholarship.

## Conflict of Interest

The authors declare that the research was conducted in the absence of any commercial or financial relationships that could be construed as a potential conflict of interest.

## Publisher's Note

All claims expressed in this article are solely those of the authors and do not necessarily represent those of their affiliated organizations, or those of the publisher, the editors and the reviewers. Any product that may be evaluated in this article, or claim that may be made by its manufacturer, is not guaranteed or endorsed by the publisher.
